# *Burkholderia pseudomallei*-absent soil bacterial community results in secondary metabolites that kill this pathogen

**DOI:** 10.1186/s13568-018-0663-7

**Published:** 2018-08-24

**Authors:** Chotima Potisap, Md Abdul Wadud Khan, Atcha Boonmee, Jorge L. M. Rodrigues, Surasakdi Wongratanacheewin, Rasana W. Sermswan

**Affiliations:** 1 Melioidosis Research Center and Department of Biochemistry, Faculty of Medicine, Khon Kaen University, 123 Mitraparb Rd, Muang District, Khon Kaen Province 40002 Thailand; 2 Department of Biology, University of Texas at Arlington, Arlington, TX USA; 3 Department of Microbiology, Faculty of Science, Khon Kaen University, Khon Kaen, 40002 Thailand; 4 Department of Land, Air, and Water Resources, University of California-Davis, Davis, CA USA; 5 Melioidosis Research Center and Department of Microbiology, Faculty of Medicine, Khon Kaen University, Khon Kaen, Thailand

**Keywords:** *Bacillus amyloliquefaciens*, *Burkholderia pseudomallei*, Metagenomics, Secondary metabolites, Soil

## Abstract

*Burkholderia pseudomallei* is a Gram-negative bacterium found in soil and the causative agent of a severe disease in humans and animals known as melioidosis. It is intrinsically resistant to many antibiotics and has been reported resistant to the drugs of choice; ceftazidime. Microbial communities in soil in the presence and absence of *B. pseudomallei* were investigated using metagenomics approach. The variation in bacterial species diversity was significantly higher in soil samples without *B. pseudomallei.* Abundances of phyla *Actinobacteria* and *Firmicutes* were found significantly higher in *B. pseudomallei*-negative soils. *Bacillus amyloliquefaciens* KKU1 in phylum *Firmicutes* was discovered from negative soil and its secondary metabolites could inhibit clinical, environmental and drug resistant isolates of *B. pseudomallei,* together with some pathogenic Gram-negative but not Gram-positive bacteria. The antimicrobial activity from KKU 1 against *B. pseudomallei* was abolished when treated with proteinase K, stable in a wide range of pH and remained active after heating at 100 °C for 15 min. Precipitated proteins from KKU1 were demonstrated to cause lysis and corrugated surfaces of *B. pseudomallei.* The minimum inhibitory concentrations and minimum bactericidal concentrations of the precipitated proteins from KKU1 against *B. pseudomallei* were 0.97 μg/ml and 3.9 μg/ml. Interestingly, Native SDS-PAGE showed small active compounds of less than 6 kDa, along with other information collectively suggesting the properties of antimicrobial peptides. For the first time, culture-independent information in melioidosis endemic area could lead to a suspected source of metabolites that may help defense against *B. pseudomallei* and other pathogenic Gram-negative bacteria.

**Electronic supplementary material:**

The online version of this article (10.1186/s13568-018-0663-7) contains supplementary material, which is available to authorized users.

## Introduction

Soil is considered to be a complex environment and is a major reservoir of living organisms either as competitive or symbiotic microbial communities (Robe et al. 2003). *Burkholderia pseudomallei* is a saprophytic Gram-negative, motile, non-spore-forming bacterium found in soil and water in endemic areas of tropical countries (Wiersinga et al. 2012). It is the causative agent of a severe infectious disease called melioidosis (Cheng and Currie 2005). The bacterium is intrinsically resistant to several antibiotics (Schweizer 2012). The drugs of choice is ceftazidime, the third-generation cephalosporins, that used to treat severe sepsis cases that have at least a 40% mortality and a vaccine is not yet available (Wiersinga et al. 2012). Importantly, a few cases of melioidosis have been reported as resistant to the drug (Schweizer 2012). Humans and animals may get infected via inhalation, ingestion but more commonly through an open wound by soil or water contaminated with the bacterium (Barnes and Ketheesan 2005). Because the bacterium can survive through the dry season perhaps by biofilm protection and then expand during a rainy season, soil is therefore the most important reservoir of the disease (Inglis and Sagripanti 2006).

*Burkholderia pseudomallei* was found unequally in soil of the endemic areas. The significant differences of some physicochemical properties of soil in the presence and absence of the bacterium have been reported (Ngamsang et al. 2015; Palasatien et al. 2008). Biological interactions of microbes in soil especially during rainy seasons, either by antagonism or mutualism, could also influence the microbial community. A similar situation was already reported, in that the phages capable of infecting *B. pseudomallei* were found mostly in the soil without this bacterium (Withatanung et al. 2016). If microbial communities in the presence and absence of *B. pseudomallei* were significantly different, soil in the endemic areas in the absence of this bacterium may be a good source to uncover organisms or compounds with inhibition or killing activity against *B. pseudomallei.*

This current study therefore analyzed and compared microbial communities in the presence and absence of *B. pseudomallei* to search for antagonistic organisms or their compounds with antimicrobial activity that could potentially be used for controlling this drug resistant *B. pseudomallei* and other pathogens in the future.

## Materials and methods

### Bacteria

Nine isolates of clinical, seven environmental, four ceftazidime resistant and six mutants of *B. pseudomallei* were obtained for the study and were kindly provided by Prof. Don Wood, Canada, and Assistant Prof. Dr. Preecha Homchampa, Khon Kaen University and five *Burkholderia thailandensis* isolates were from the Melioidosis Research Center, Khon Kaen University. Three *Burkholderia mallei* isolates were kindly provided by Prof. Sumalee Tungpradubkul, Mahidol University, Thailand. *Enterococcus* sp.*, Streptococcus pneumonia, Stenotrophomonas maltophilia, Klebsiella pneumonaie, Acinetobacter baumannii, Salmonella* group D*, Pseudomonas aeruginosa, Shigella* group D*, Vibrio parahaemolyticus, Escherichia coli* and *Proteus vulgaris* were obtained from Srinagarind hospital, Faculty of Medicine, Khon Kaen University. All of bacterial strains including *Bacillus amyloliquefaciens* KKU1 (MRCKKU84) were deposited in the culture collection belonging to World Data Centre for Microorganisms (WDCM) (Registration Number 1130).

### Soil sampling and culture

Fifty soil samples were taken a few weeks after rainy season from a 2 km^2^ area belonging to the Faculty of Agriculture, Khon Kaen University, Khon Kaen province in the northeast of Thailand. The sampled area was composed of grasses and small shrubs and had been left uncultivated for several years. Soil samples were randomly collected at a 15-cm depth and kept tightly sealed in plastic bags in the dark at ambient temperature until they were transported to the laboratory. Microbes in soil samples were cultured on the Ashdown’s selective medium and enrichment method the next day to identify the presence or absence of *B. pseudomallei* (Limmathurotsakul et al. 2012; Wuthiekanun et al. 1990). The positive soil samples for *B. pseudomallei* were counted from both direct and enrichment cultures followed by confirmation of suspected colonies by the latex agglutination test (Samosornsuk et al. 1999). The negative soil samples were determined to be negative by direct and enrichment cultures and also semi-nested PCR detection.

### Detection of *B. pseudomallei* in soil

Total DNA was extracted from each soil sample using the PowerSoil^®^ DNA isolation kit (MO-BIO Laboratories, Carlsbad, CA, USA) according to the manufacture’s recommendations and was used to amplify the 16S–23S spacer gene by semi-nested PCR that was specific for *B. pseudomallei* (Merritt et al. 2006). The sensitivity of the method to detect *B. pseudomallei* DNA in soil was evaluated by spiking to obtain 0.1–50 ng of *B. pseudomallei* DNA in the DNA solution that was extracted from 1 g soil. Six microliters from each spiking amount were used for the semi-nested PCR.

### Physicochemical properties of soil

The pH, total organic carbon (TOC), total nitrogen (TN), TOC:TN (C:N ratio), exchangeable calcium (EC) and extractable iron (EI) in each soil sample were analyzed as previously described (Ngamsang et al. 2015).

### Metagenomics approach for analyzing bacterial community in soil

Owing to the astonishing diversity of microorganisms present in soils and the fact that approximately only 1% of them could be cultured in laboratory conditions (Amann et al. 1995), a culture-independent method to identify and compare microbial communities in soil with and without *B. pseudomallei* was used.

Six positive and three negative soil samples were randomly selected for the study and the data from six positive replicates were analyzed and compared with three negative replicates. Community diversity and similarities were calculated by using both taxonomic and phylogenetic measurements. The taxonomic similarity was calculated as the proportion of shared operational taxonomic units (OTUs), whereas phylogenetic similarity was calculated as the proportion of shared phylogenetic branch lengths between communities.

#### Soil DNA extraction

The total DNA was extracted, quantified, diluted, and stored in a − 20 °C freezer until used. All soil DNA samples were displayed on a 0.7% Tris–Acetate-EDTA agarose gel and stained with ethidium bromide to verify their integrity.

#### Pyrosequencing of 16S rRNA gene

DNA extracted from soil samples was used for the barcoding of the primers for the pyrosequencing (Teixeira et al. 2010). PCR amplification of the hypervariable V4 region of the 16S rRNA gene was performed using the eubacterial primers 563F and 802R as previously described (Teixeira et al. 2010). Equimolar amplicon suspensions obtained from the PCR amplification were subjected to pyrosequencing using a Genome Sequencer FLX system (454 Life Sciences, Branford, CT) at the Michigan State University Genomics Technology Support Facility, East Lansing, MI, USA. The sequence data was deposited under GenBank SRA database: SRP136447.

#### Sequence analyses and diversity metrics

Sequence processing and analyses were conducted using the open-source bioinformatics pipeline Quantitative Insights Into Microbial Ecology (QIIME) version 1.8.0 (Caporaso et al. 2010). Following the removal of raw sequences with anonymous bases, the assignment of OTUs was performed on the quality-filtered sequences using the RDP database (Wang et al. 2007). The algorithm search (Edgar 2010), which has an additional power of detecting chimera sequences, was used for OTU-picking based on a de novo approach at a minimum of 97% sequence identity. Only sequences that passed the quality filter were used for downstream analyses. A randomized selection of 2000 sequences per sample was used for rarefaction. The process was repeated 10 times and results are based on the means of these 10 trials. Sequences were assigned to operational taxonomic units (OTUs) based on 97% DNA identity using a de novo OTUs-picking protocol. Alpha diversity was calculated using both phylogenetic (PD) and taxonomic (Shannon index) metrics (Faith 1992; Shannon 1948). Beta diversity was calculated using the Bray–Curtis index for taxonomic dissimilarity and weighted by UniFrac for phylogenetic dissimilarity (Lozupone and Knight 2005).

The strict definition of Whittaker’s (1975) for alpha diversity as the average richness across all soil samples and for beta diversity as variation in species diversity between samples was used. Sampling was not designed to take into consideration the directional turnover along a gradient of beta diversity (Anderson et al. 2011), owing to patchiness of *B. pseudomallei* presence in soil.

#### Statistical analyses

The program SPSS, version 16.0 was used to analyze the physicochemical properties of *B. pseudomallei*-positive and negative soils. The independent samples *t* test was used when two separate sets of independent data were normally distributed. The non-parametric test, Mann–Whitney U test, was used when two separate sets of independent data were not normally distributed. A nonparametric test was also used to compare the CFU of *Bacillus* spp. in positive and negative soil samples.

Alpha diversity values between soil samples were compared using a non-parametric two-sample *t*-test with 999 Monte Carlo permutations. To compare the relative abundances of major phylum-level taxa between samples, a non-parametric *t*-test was carried out based on a bootstrap procedure with 100 permutations. An analysis of the similarities (ANOSIM) test was performed with 99 permutations to check whether soil samples harbored significantly different microbial communities (Clarke 1993). All the statistical analyses were conducted in the QIIME 1.8.0 environment.

### Isolation of *Bacillus* spp. from soil

*Bacillus* spp. were cultured from soils that were found to be positive and negative for *B. pseudomallei* using the method as described by Travers (1987) with some modifications. One gram of soil was mixed with 10 ml sterile distilled water and boiled for 5 min. The supernatant was tenfold diluted and 100 μl was used to spread on a nutrient agar plate and incubated at 37 °C for 18–24 h. Bacterial colonies with a morphology of being white and large with wavy, lobed margins like *Bacillus* spp. were sub-cultured and confirmed by Gram’s stain.

### Screening for the production of antimicrobial compounds against *B. pseudomallei*

A single colony of each *Bacillus* isolate was grown in Luria–Bertani (LB) broth at 37 °C for 72 h and centrifuged at 14,600×*g* for 10 min (Avanti^®^ J**-**E, Beckman Coulter, Brea, CA) to obtain the supernatant. The agar-well diffusion method was used to determine antimicrobial activity in the supernatant (Umer et al. 2013). *B. pseudomallei,* as a test organism, was grown in Luria–Bertani (LB) broth to reach the log phase and 100 μl solution was spread on Mueller–Hinton agar (MHA) plates. The plates were dried and punched using a sterile micropipette tip to obtain 4–6 wells per plate. One hundred microliters of supernatant from each *Bacillus* spp. were then added into each well and incubated at 37 °C for 24 h. Clear inhibition zones against *B. pseudomallei* were observed to indicate antimicrobial activity. Ceftazidime, the drug of choice for *B. pseudomallei* was used as a positive control and the production medium was used as a negative control.

### Species identification

Four *Bacillus* spp. from soil with inhibition activity against *B. pseudomallei* were identified using DNA sequencing in the 16S rDNA region. Genomic DNA from each isolate was extracted using a gDNA extraction RBC kit (RBC ribosicen, Taiwan) and amplified using specific primers and conditions that were specific to *Bacillus* spp. as previously described (Ghribi et al. 2012). The PCR products were sequenced (First Base laboratories Sdn Bhd, Malaysia) and the nucleotide sequences were used for species identification using the Blast program (https://blast.ncbi.nlm.nih.gov/Blast.cgi?PAGE_TYPE = BlastSearch).

### The spectrum of antimicrobial activity

A single colony of each *Bacillus* isolate was grown in minimal medium supplemented with 1% glucose (Jamil et al. 2007) and incubated at 37 °C for 72 h to represent secondary metabolites. Bacterial cultures were centrifuged at 14,600×*g* for 10 min (Avanti^®^ J**-**E, Beckman Coulter, Brea, CA) and the supernatant representing secondary metabolites were used to test for their activity or kept at − 20 °C until being used. One hundred microliters of the metabolites were used to determine the antimicrobial activity against *B. pseudomallei, Burkholderia* spp*., B. pseudomallei* mutants and other Gram-negative and Gram-positive pathogens by the agar-well diffusion method (Umer et al. 2013).

### Partial characterization of the antimicrobial compounds

#### Stability

For heat stability, 1 ml of the 72 h metabolites from each isolate was put in 1.5 ml micro-centrifuge tubes and were incubated at either 25, 40, 60 or 100 °C for 15, 30 or 45 min using a heating block (Major Science, Saratoga, CA). For pH stability, the pH of metabolites were adjusted to have a pH range from 2 to 14 using 1 N HCl or 1 N NaOH and left at 4 °C overnight. Prior to assessing for the antimicrobial activity by the agar-well diffusion method, the pH was readjusted to pH 7.0. For proteolytic digestion, Proteinase K (Amresco LLC, OH) was used to digest proteins in the metabolites for 4–30 min according to the manufacturer’s instructions and then the enzyme was inactivated and the antimicrobial activity measured using the agar-well diffusion method.

#### Production of the antimicrobial compounds

*Bacillus amyloliquefaciens* KKU1 was cultured in duplicate in 150 ml of Luria Bertani (LB) broth at 37 °C with 200 rpm shaking. One milliliter of the culture was taken and used to measure OD at 600 nm at intervals for 96 h. The culture supernatant from each time point was also used to test for the antimicrobial activity against *B. pseudomallei* using the agar-well diffusion method.

#### The effect of the antimicrobial compounds on cell surface

*Burkholderia pseudomallei* and *E. coli* were grown in LB medium to obtain a cell density of 10^8^–10^10^ CFU/ml. Fifty microliters of the bacterial cell suspension were incubated with 30 µg/µl of precipitated proteins from *B. amyloliquefaciens* KKU1 for 24 h and 10 µl were placed on a 0.20 µM pore-size membrane filter (Schleicher & Schuell, Dassel, Germany) and then processed for EM image observations (Hartmann et al. 2010). EM images were taken using a HITACHI S-3000 N 55 microscope (Hitachi High technology, Japan) at electron energies between 10 and 20 kV.

#### Partial purification of the antimicrobial compounds

A single colony of *B. amyloliquefaciens* KKU1 was grown in 500 ml M9 minimal medium (Cold Spring Harbor protocol) supplemented with 1% glucose and incubated at 37 °C for 72 h before the supernatant was collected by centrifugation at 14,600×*g* for 10 min (Avanti^®^ J**-**E, Beckman Coulter, Brea, CA). The supernatant was precipitated by 40–80% saturated (NH_4_)_2_SO_4_ at 4 °C and then solubilized with Tris-buffer before being dialyzed against Tris-buffer, pH 7.5 at 4 °C for 24 h. The percentage that gave highest anti-microbial activity against *B. pseudomallei* was used for a large-scale preparation of the proteins. The concentrations of precipitated proteins were determined by the Bradford technique (Bio-Rad Laboratories, Inc., PA).

#### Effect of the precipitated proteins on *B. pseudomallei*

The precipitated proteins from culture supernatant of *B. amyloliquefaciens* KKU1 isolates were filtered through 0.2 µm membranes, adjusted the concentration to 0.025 mg/ml and then used to determine the minimum inhibitory concentration (MIC) and minimum bactericidal concentration (MBC) by micro-broth dilution (Hoelzer et al. 2011). In brief, the antimicrobial compounds were diluted in 96-well plates by two-fold serial dilutions using MHB. An inoculum dose of 10^5^–10^6^ CFU/ml of *B. pseudomallei* then was added into each well, mixed gently and then incubated at 37 °C for 24 h. The last concentration that provided a clear solution when compared to the growth control was recorded as the MIC. The MBC was evaluated by pipette from each dilution of the clear wells, diluting with PBS pH 7.2 and then 10 µl of each dilution was dropped onto Ashdown’s agar for colony counts. Bacterial cells from the last turbid well were collected and stained with Gram’s stain.

#### Native-PAGE and SDS-PAGE of crude precipitated proteins from *B. amyloliquefaciens* KKU1

Thirty micrograms of precipitated proteins from culture supernatants of *B. amyloliquefaciens* KKU1 were separated in duplicate using 15% Native-PAGE (Barboza-Corona et al. 2007). The proteins were separated in duplicate for staining with Coomassie blue and tested for inhibition activity by placing on an LB agar plate spread with *B. pseudomallei* and incubated at 37 °C for 24 h. The protein bands with activity were observed as having clear inhibition zones on a *B. pseudomallei* lawn. The active protein bands were cut and eluted by 50 mM Tris–HCL pH 8.0 at room temperature, overnight. The proteins were separated in 15% SDS-PAGE and stained by the silver staining method (Meng et al. 2012).

## Results

### Soil sampling, culture and physicochemical factors of soil

Twenty-seven soil samples were found to be positive for *B. pseudomallei*, 24 by direct culturing on Ashdown’s selective medium and three by an enrichment method. The colony counts from direct culture varied from 1 × 10^3^ to 6.6 × 10^6^ colonies/g of soil. For the negative soil site, 23 samples were negative by both direct and enrichment cultures. Two samples, however, were found positive using the semi-nested PCR and were then excluded. The average colony count in positive soil samples was 2.4 × 10^5^ colonies/g of soil.

The pH, TN and EI were factors that were significantly different between positive and negative soil samples. The average pH in both positive and negative soil samples was in the acidic range. The average pH in the positive soil was 4.3 (4.0–4.6), while the negative soil was 3.9 (3.7–4.2) (*p* < 0.05). For EI, the positive soil had a higher average EI of 45.4 ppm (16–68 ppm) in comparison to 17.3 ppm (11–25 ppm) observed in negative soil samples (*p* < 0.05). The average of TN in positive soil was 0.03% (0.025–0.032%) and negative soil was 0.02% (0.018–0.028) (*p* < 0.05).

### Metagenomics approach for analyzing bacterial community in soil

Over 5000 bacterial sequences per sample were detected and 1000–1400 OTUs were associated with the rarefaction curves calculated with Distance-Based OTUs that showed different patterns between positive and negative soil samples. Soil samples without *B. pseudomallei* showed higher diversity than positive soil samples (Additional file 1: Figure S1).

**Fig. 1:**
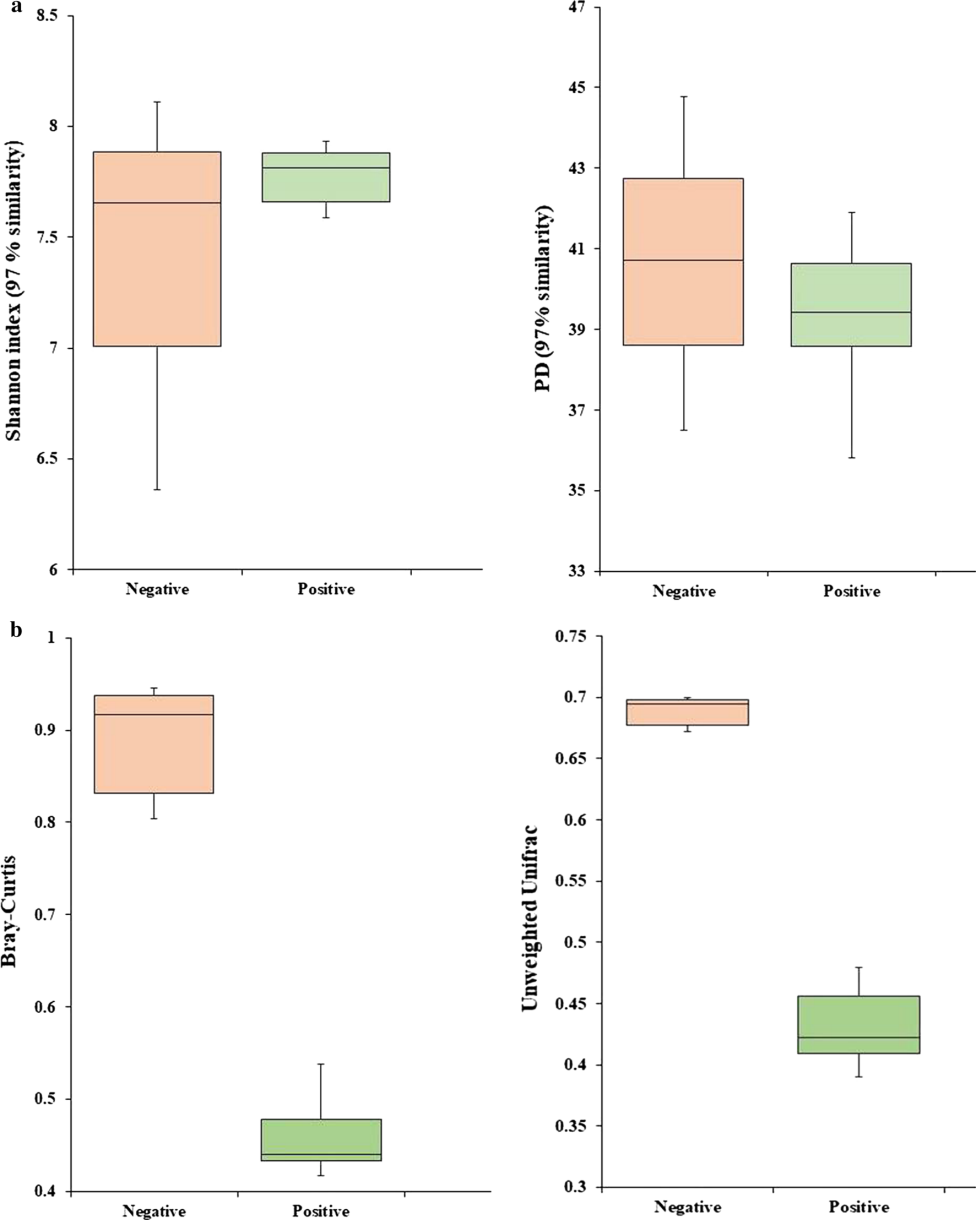
The comparison of alpha- and beta-diversity between negative and positive soils. **a** Alpha-diversity (Shannon index and Faith’s Phylogenetic Diversity) and **b** beta-diversity (Bray–Curtis and unweighted Unifrac distance matrix)

There were no measured differences in alpha diversity of observed species between positive and negative soils (Fig. 1). Both estimates at taxonomic (Shannon index) and phylogenetic (Faith’s Phylogenetic Diversity) levels using a 97% identity value for the 16S rRNA gene were similar. In contrast, the beta diversity was significantly higher in soils without *B. pseudomallei.* The community similarities as measured both phylogenetic (UniFrac) (*t* = − 4.1, *p *< 0.001) and taxonomically (Bray–Curtis) (*t* = − 4.68, *p *< 0.001), were significantly different (Fig. 1).

**Fig. 2:**
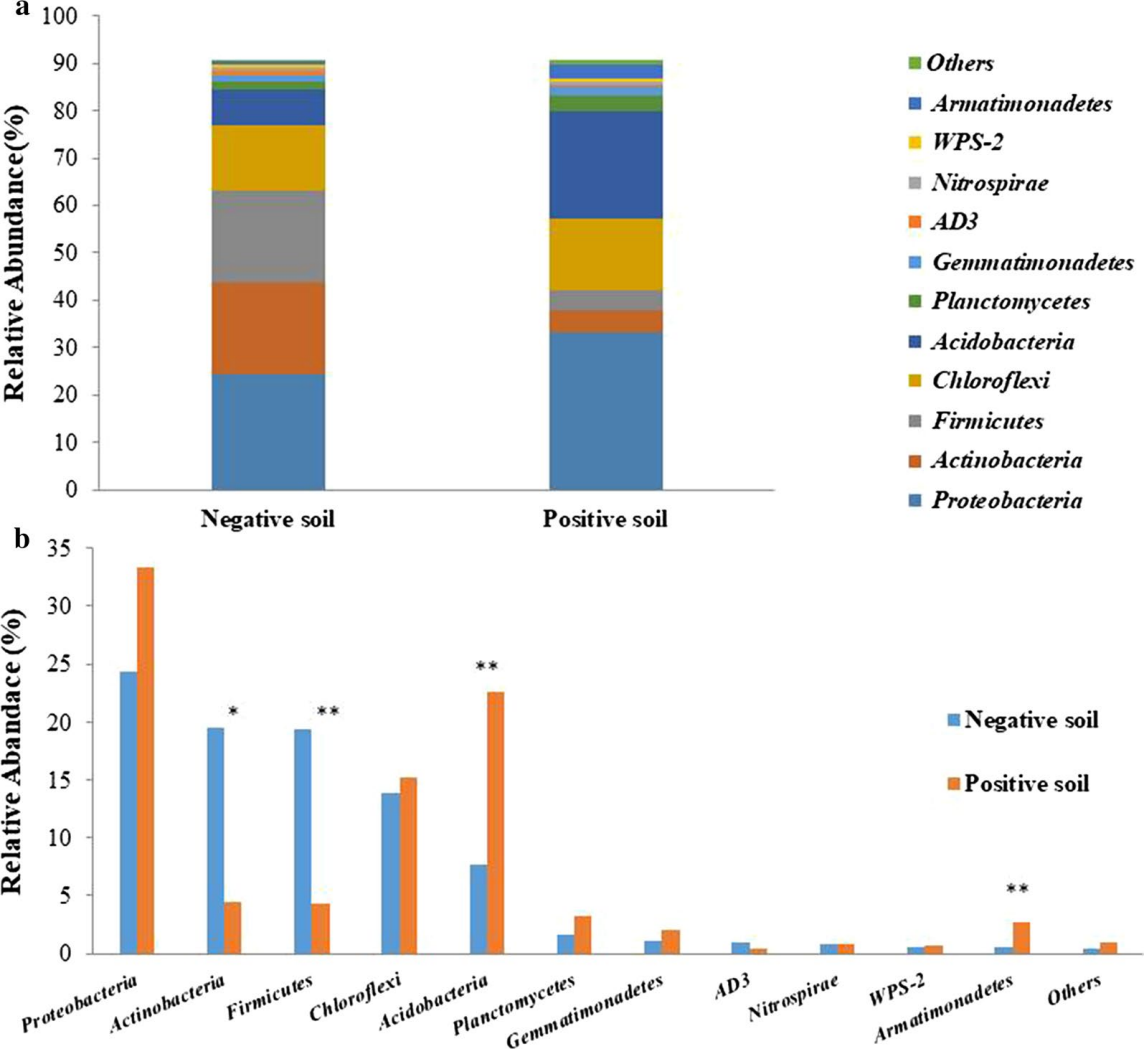
The comparison of microbial composition in positive and negative soils. **a** The stack graph of microbes in major phyla. **b** The comparisons of each major phyla. The blue bars represent negative soil and orange bars represent positive soil. Asterisks (*p < 0.05, **p < 0.001) indicate the taxa that were significantly different in relative abundances of negative and positive soil samples

There were 11 phyla of the Domain *Bacteria* identified in soil samples associated with the presence and absence of *B. pseudomallei*, of which the major phyla were *Proteobacteria, Actinobacteria, Firmicutes, Chloroflexi, Acidobacteria, Planctomycetes Gemmatimonadetes*, *AD3*, *Nitrospirae*, *WPS*-*2*, and *Armatimonadetes* (Fig. 2a). A non-parametric t test was carried out in order to compare the frequencies of major bacterial taxa between negative and positive soil samples using a bootstrap procedure with 100 permutations. Results showed the ratios of phyla *Acidobacteria* (22.57%) and *Armatimonadetes* (2.69%) over other phyla in the positive soil samples were significantly higher than those present in negative soil samples (*p* < 0.01) (Fig. 2b). On the other hand, *Actinobacteria* (19.5%) and *Firmicutes* (19.4%) were the major phyla significantly higher in relative abundances in the negative soils when compared to those values observed for positive soil samples (*p* < 0.05 and *p* < 0.01) (Fig. 2b).

Next, comparisons between negative and positive soil samples at bacterial class levels from nine phyla showed the presences of class *Bacilli* (*p* < 0.01) and *Actinobacteria* (*p* < 0.05) were significantly higher in the negative soils than the positive ones (Additional file 2: Figure S2). Class *Bacilli* was increased from 3.82% (± 0.82; 95% CI) in the positive to 18.75% (± 18.29; 95% CI) in the negative soils and *Actinobacteria* was increased from 1.82% (± 0.58; 95% CI) in the positive to 16.7% (± 20.71; 95%CI) in the negative soils. In contrast, the largest population that increased in response to the presence of the pathogen was *TM1* (*Phylum Acidobacteria*), which increased from 3.33% (± 3.06; 95% CI) in negative soils to 8.08% (± 1.81; 95% CI) in positive soils, followed by *Acidimicrobiia* (*Phylum Actinobacteria*) that increased from 2.05% (± 2.15; 95% CI) to 7.59% (± 1.15: 95% CI), and *Solibacteres* (*Acidobacteria*) increased from 1.13% (± 1.36; 95% CI) to 3.45% (± 0.49; 95% CI) (Additional file 3: Figure S3).

### *Bacillus* spp. isolation and species identification

The class *Bacilli* was the largest proportion that significantly increased in the negative soil and the isolation of them from soil was expected to obtain a source of antimicrobial compounds. Sixty-Six *Bacillus* spp. were able to be isolated from both positive and negative soils, of which 68% (45/66) were from the negative soils. All isolates were tested for antimicrobial activity against *B. pseudomallei* and six isolates that gave clear zones on *B. pseudomallei* lawn were obtained only from the negative soil. Two of them (MRCKKU72 and MRCKKU73) were characterized (Boottanun et al. 2017) and other four, named as KKU1 (MRCKKU84), KKU3 (MRCKKU85), KKU11 (MRCKKU86) and KKU14 (MRCKKU87), are reported here together with diversity of microbes in the presence and absence of *B. pseudomallei*. PCR amplification at the 16S rDNA region of DNA from these isolates to obtain 1500 bp products were analyzed for their nucleotide sequences. The comparison of 16S rDNA sequences from four isolates (GenBank Accession number of KKU1, KKU3, KKU11 and KKU14 are BankIt2097833 SequenceKKU1 MH114079, BankIt2097833 SequenceKKU3 MH114080, BankIt2097833 SequenceKKU11 MH114081 and BankIt2097833 SequenceKKU14 MH114082) with the GenBank database revealed nucleotide similarities of 99.8 or 99.9% with *B. amyloliquefaciens* (The accession number that matched to the GenBank Accession Number was NC_014551.1).

### Spectrum of the antimicrobial activity

The metabolites from KKU 1, 3, 11 and 14 were tested against *B. pseudomallei* and *Burkholderia* spp. (Table 1, Additional file 4: Table S1). KKU 1 and 14 showed broader spectrums of inhibition than others. They could inhibit 50% of drug resistant isolates, some clinical (18–45%) and almost all of the environmental isolates (71–100%) but not *B. thailandensis*, a closely related bacterium nor *B. cepacia*. Interestingly, KKU1, 3 and 14 could inhibit 50% of *B. mallei* which is an important pathogen of the horse that also could infect humans. For mutants and their wild types, the capsule and LPS (O-side chain) mutants were resistant to the metabolites from the four isolates while their wild types were sensitive. Another set of biofilm mutants was vice versa as the wild type showed resistance to the metabolites from all isolates while its mutants were sensitive to KKU1, 3 and 14 (Table 1, Additional file 5: Table S2).

**Table 1:** Antimicrobial activities of culture supernatants from *B. amyloliquefaciens* against *Burkholderia* spp

Bacterial indicators	KKU1	KKU3	KKU11	KKU14
*Bp* clinical isolates (9)	2	1	3	5
*Bp* environmental isolates (7)	7	5	0	7
*Bp* CAZ resistance strains (4)	2	1	1	2
*Bp* mutant strains				
1026b wild type	Y	Y	Y	Y
SR1015 capsule mutant	N	N	N	N
SRM117 O-side chain mutant	N	N	N	N
MM53 flagellin mutant	Y	Y	N	Y
H777 wild type	N	N	N	N
M10 biofilm mutant	Y	Y	N	Y
M6 biofilm mutant	Y	Y	N	Y
*B. thailandensis* (5)	0	0	0	0
*B. cepacia* (4)	0	0	0	0
*B. mallei* (3)	2	2	0	2

Numbers in brackets indicate number of test isolates

For mutant results, they were placed under their wild type. *Y* sensitive, *N* resistant

When the metabolites from KKU1 and 14 were selected to test against Gram-positive and Gram-negative pathogens, they could inhibit all Gram-negative bacteria that were tested except *K. pneumoniae* and *A. baumannii* but not Gram-positive (Additional file 6: Table S3).

### Effects of proteinase K, temperature and pH on antimicrobial activity

The antimicrobial activity from KKU1 was abolished when treated with proteinase K while activities from the other three were partially lost. As antimicrobial peptides were of primary interest, KKU1 was then selected for further study. The antimicrobial activity from KKU1 metabolites was stable when heated at 25–80 °C for 15–45 min and 100 °C for 15 min; then activity was lost when heated to 100 °C for 30 min. They were stable in a wide range of pHs from 4 to 8 and partially lost at pH 2 and pH 12 (Additional file 7: Table S4).

### The production of secondary metabolites with antimicrobial activity

**Fig. 3:**
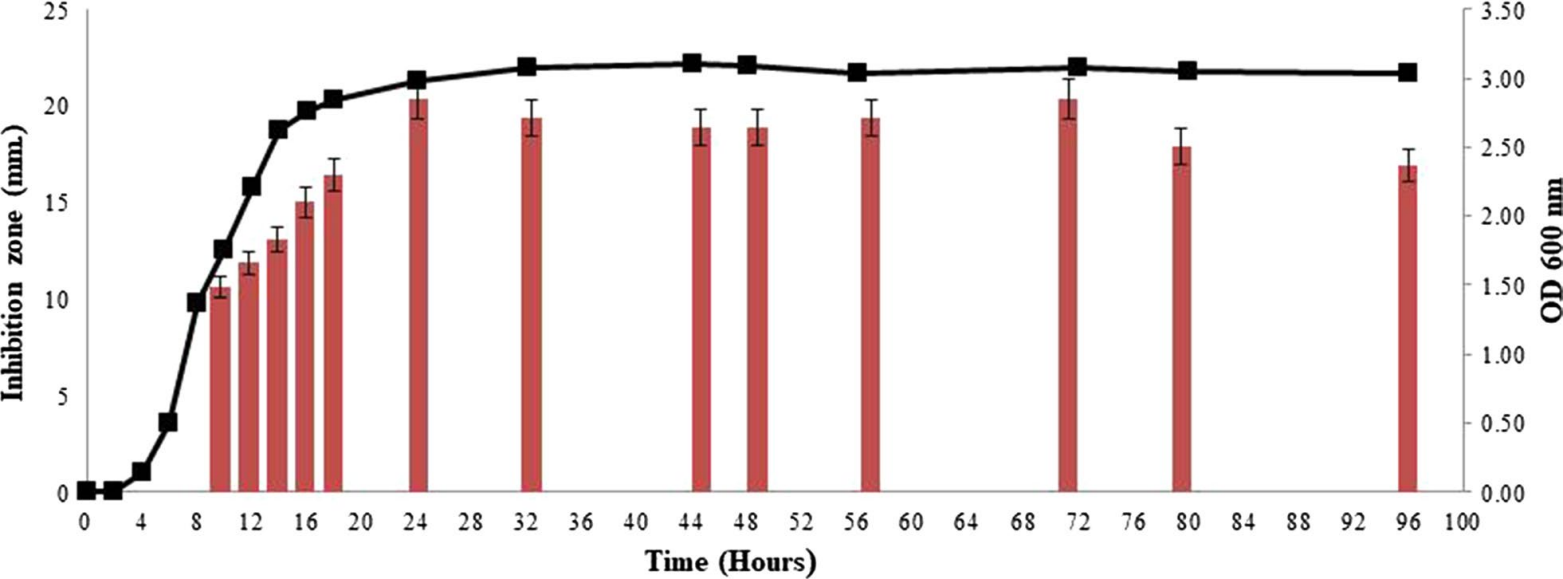
The production of secondary metabolites active against *B. pseudomallei* from *B. amyloliquefaciens* KKU1 in a time course manner. The production of secondary metabolites displayed as sizes of the inhibition zones in nm against *B. pseudomallei* are plotted on the left Y axis and growth curve as measured by OD at 600 nm are plotted on the right Y axis. The X axis represents the time in hours

The metabolites from *B. amyloliquefaciens* KKU1 showed inhibition activity against *B. pseudomallei* after being cultured for 10 h. The highest inhibition activity was observed at 24–72 h when the KKU1 entered the stationary growth phase with an inhibition zone of 21 mm (Fig. 3).

### Partial purification of secondary metabolites and their killing activities

The concentration of precipitated proteins from the culture supernatant of KKU1 was 0.15 mg/ml. The minimum inhibitory concentration (MIC) and minimum bactericidal concentration (MBC) of the precipitated proteins from KKU1 against *B. pseudomallei* were 0.97 μg/ml and 3.9 μg/ml. Ceftazidime, as a positive control, had an MIC and MBC equal to 2 μg/ml and 4 μg/ml. The precipitated proteins from KKU1 inhibited *B. pseudomallei* at a lower concentration than ceftazidime and could kill the bacterium using similar concentrations.

**Fig. 4:**
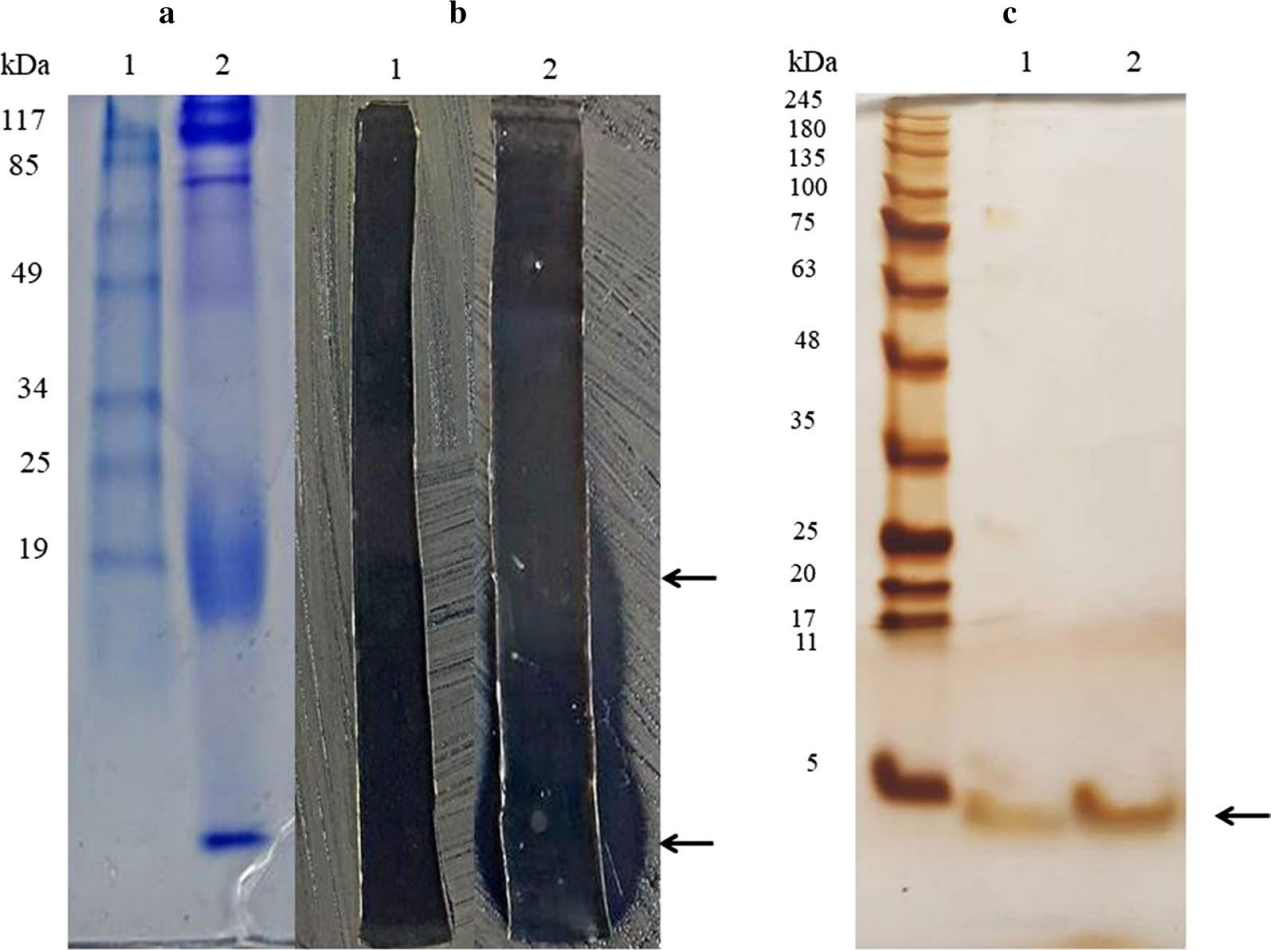
Gel electrophoresis of crude proteins with antimicrobial activity from KKU1. **a** Native-PAGE stained with Coomassie Brilliant blue of (1) size marker and (2) the crude proteins from culture supernatant of *B. amyloliquefaciens* KKU1. **b** The gel strip of control (1) and precipitated proteins from Native-PAGE (2) that were placed on *B. pseudomallei* lawns show clear inhibition zones from the action of active compounds. **c** The SDS-PAGE staining by silver stain of extracted proteins from clear zone positions on Native-PAGE (1) from the lower arrow and (2) from the upper arrow

Precipitated proteins from KKU1, when separated in Native-PAGE and stained with Coomassie Brilliant Blue (Bio-Rad Laboratories Ltd, CA) showed a thick broadened band of approximately 19 kDa and at the dye front (Fig. 4a). Both of them showed clear zones on *B. pseudomallei* lawn (Fig. 4b). When proteins from clear zone positions were extracted, separated on SDS-PAGE and stained with silver staining, a band of less than 6 kDa and a thin band at the 19 kDa position can be detected (Fig. 4c).

### Bacterial morphology under scanning electron microscope

**Fig. 5:**
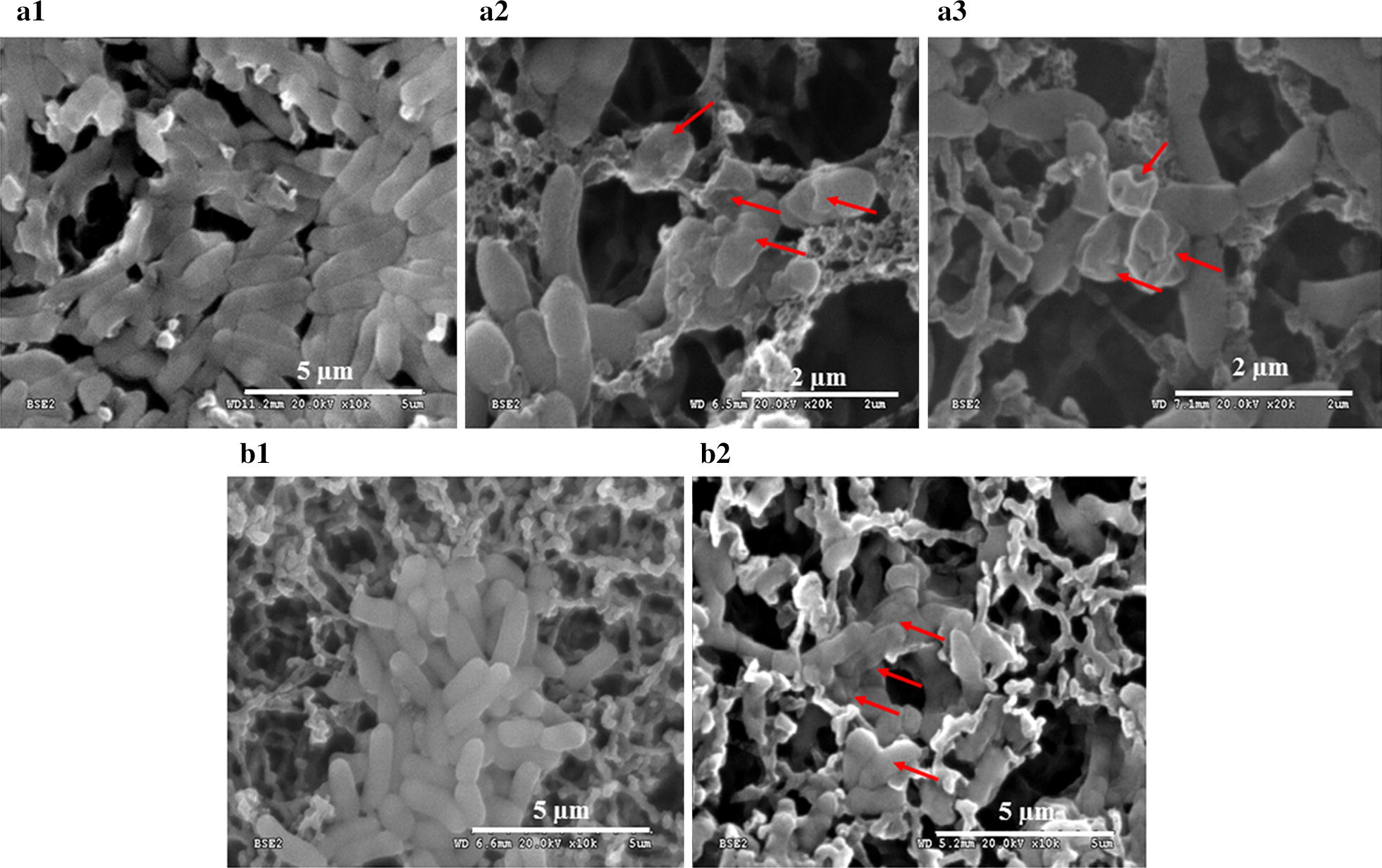
Scanning electron microscopy shows the effects of *B. amyloliquefaciens* metabolites on *B. pseudomallei* and *Escherichia coli*. **a1** *B. pseudomallei* control, **a2**–**a3** *B. pseudomallei* treated with precipitated proteins from culture supernatants of *B. amyloliquefaciens*. Arrows point to damage of bacteria cells. **b1** *E. coli* control and **b2** *E. coli* treated with precipitated proteins from culture supernatant of *B. amyloliquefaciens*

The untreated *B. pseudomallei* and *E. coli* cells observed under SEM microscopy in standard LB medium appeared as 2–3 µm long cells with smooth and intact surfaces (Fig. 5a1, b1). The bacterial cell surfaces that were treated with precipitated proteins for 24 h showed corrugated surfaces with a somewhat dimpled skin appearance but the average length remained unaltered (Fig. 5a2–3, b2).

## Discussion

A majority of bacterial pathogens that contributed to fatal infections were predicted by the Infectious Disease Society of America to be resistant to at least one of the antibiotics usually used for the treatment of bacterial infections (Hassan et al. 2012). Melioidosis was found to be difficult to treat as the bacterium is intrinsically resistant to several antibiotics and also resistant to its drug of choice, even though rarely (Schweizer 2012). The metagenomics approach was used in this study therefore, not only to explore the diversity and ratio of microbes in each habitat, but the possibility of obtaining a source of compounds that could be active against *B. pseudomallei*.

A few abiotic factors have been reported to be significantly different when compared between soils that were positive and negative for *B. pseudomallei* (Ngamsang et al. 2015; Palasatien et al. 2008). The factors investigated here were also significantly different in these two groups of soils. It should be noted also that the average pH values were all in the acidic range that was suitable for *B. pseudomallei*. The C:N ratio of positive soils was found to be lower than the negative soils that favored the decomposition. *B. pseudomallei* may benefit from this condition as members of the genus *Burkholderia* are known to be adapted to soil, having large genomes and capable of utilizing multiple C sources (Coenye and Vandamme 2003). It is well known that not only physicochemical properties of soil may influence the diversity and abundance of living organisms, but also biological interactions occurring between different groups of organisms may contribute to the presence of a specific taxon (Anderson et al. 2011; Whittaker 1975). The microbial populations obtained from randomly selected samples for analyses in this study then were affected by both the environmental niche and microbial interactions that provided existing populations as was cross-sectionally observed in this study.

In this study, while alpha diversity indices (the mean species diversity), both at taxonomic and phylogenetic levels, were similar for positive and negative soils, the beta diversity indices (diversity within habitats) were significantly higher for negative soils. These results suggested that the overall number of microbial taxa in soil is not an important factor in order to limit the presence of *B. pseudomallei*, but the composition of the microbial community may influence this human pathogen success when colonizing the soil environment.

In this study, 11 major phyla were detected in both *B. pseudomallei* positive and negative soil samples. The relative abundances, however, were significantly different for a few groups and the first two phyla, namely *Actinobacteria* and *Firmicutes*, were significantly higher in negative in comparison to positive soils. Members in these phyla were known as having potential to secrete antimicrobial agents such as polyketines and antimicrobial peptides that may inhibit *B. pseudomallei* (Sansinenea and Ortiz 2011). The phylum *Actinobacteria* is characterized as Gram-positive bacteria that typically found in the soil, playing an important part of decomposition of organic matter (Janssen et al. 2002). Members of this group, such as the genus *Streptomyces*, contribute to production of several antibiotics that are important in medicine including aminoglycosides, anthracyclines, chloramphenicol, macrolide, and tetracyclines (Qin et al. 2009, 2011). *Streptomyces* and other *Actinomycetes* are major contributors to biological buffering of soils and play roles in organic matter decomposition conductive to crop production (Suela Silva et al. 2013). The other phylum is the *Firmicutes*, Gram-positive bacteria with a low% G + C content (less than 50%) and constitutes one of the main phyla within the *Bacteria* with highly diversity in both morphology and lifestyle. In this current study, the relative abundance of members of the class *Bacilli* were highly increased in negative soils; *Bacillus* species producing a large number of antimicrobial peptides that could be used as biocontrols for plant diseases (Sansinenea and Ortiz 2011). Specifically, a *Bacillus* sp. was reported to degrade quorum sensing signal molecules of the *N*-acylhomoserine lactone (AHLs) in *B. pseudomallei* culture supernatants and, therefore, could decrease biofilm formation (Ramli et al. 2012). Two biosurfactants from *Bacillus subtilis* and *Bacillus licheniformis* also have been reported to have specific anti-adhesion activities and selectively inhibit biofilm formation of two pathogenic strains (Wu et al. 2005).

In this study, it was also observed that the relative abundances of phyla *Acidobacteria*, and *Armatimonadetes* were significantly higher in positive soils when compared to negative soils. The phylum *Acidobacteria* is one of the most dominant phyla in soil microbial communities, suggesting that they play an important role in this ecosystem (Lee et al. 2008). This group, however, is very difficult to grow in laboratory conditions and information about their potential metabolic functions in soils is still very limited (Davis et al. 2011). The presence of *Acidobacteria* group in the positive soils is correlated with the acidic environment found in the presence of *B. pseudomallei*. Whether the bacteria in this phylum highly contribute to the presence of *B. pseudomallei* requires further investigation.

*Bacillus* species can produce structurally diverse secondary metabolites, which exhibit a wide spectrum of antibiotic activity. These are most commonly known as antimicrobial peptides (AMPs) and are promising alternatives for a new generation of antibiotics against bacteria, fungi and even viruses (Sumi et al. 2015). In the present work, four *B. amyloliquefaciens* strains produced their secondary metabolites that were active against both clinical and environmental isolates of *B. pseudomallei* and also *B. mallei,* which is an obligate mammalian pathogen and closely related to *B. pseudomallei.* They could also inhibit most of the drug resistant isolates (3/4, 75%). KKU1, 3 and 14 showed more inhibition against environmental (70–100%) than clinical isolates (10–50%). *B. amyloliquefaciens* acting antagonistically against *B. pseudomallei* in soil that supports the success of isolation of *B. amyloliquefaciens* from soil samples that were negative for *B. pseudomallei*. Analysis of genomic islands of *B. pseudomallei* could indicate the differences between clinical and environmental isolates (Bartpho et al. 2012). Any contribution from genes found more commonly in the environmental isolates in these regions to the greater susceptibility of environmental isolates to the metabolites of *B. amyloliquefaciens,* however, needs to be confirmed.

The 16S rDNA sequences from four *B. amyloliquefaciens* isolates were matched to the same accession number in the GenBank database and the inhibition profiles against other Gram-negative and positive of these four *B. amyloliquefaciens* isolates were similar. Their inhibition profiles against a number of *B. pseudomallei* isolates, however, were different. Therefore, these isolates should remain recognized as different and do not produce the same compounds. They are also different from two isolates that were previously reported from this current group (Boottanun et al. 2017) by observing their inhibition profiles. Restriction enzymes digestion patterns of KKU1, 3 and 11 were similar but different from KKU14 (data not shown).

The metabolites tested against *B. pseudomallei* mutants and their wild types showed the capsule and LPS (O-side chain) mutants were resistant to the metabolites from the four isolates while their wild types were sensitive. Gram-negative bacteria were reported to have several mechanisms to inhibit natural antimicrobial peptides (AMPs) such as proteolytic degradation, barriers caused by bacterial cell envelopes, such as capsule polysaccharides, biofilm-forming exopolysaccharides, and the *O*-polysaccharides of LPS or the function of the efflux pump (Gruenheid and Le Moual 2012). These barriers were suspected to function as a charge difference as polysaccharides composed of LPS and the capsule had an increased negative charge relative to the outer membrane of the bacteria (Palffy et al. 2009) while AMPs are usually positively charged (Sumi et al. 2015). Capsule and LPS mutants of *B. pseudomallei* were resistant to *B. amyloliquefaciens* metabolites but not the flagellin mutant while their wild type was not; the charge or structure of these antimicrobial compounds may be different from what has been reported or their action on the target membrane may be different that elimination of LPS or capsule helps facilitate their action. *B. thailandensis* is a non-pathogenic organism also found in soil and closely related to *B. pseudomallei* but was not affected by these metabolites and, has been reported to contain a different LPS structure from *B. pseudomallei* (Novem et al. 2009). It is therefore interesting to investigate if this difference plays any role in the resistance to these *B. amyloliquefaciens* metabolites. In the case of biofilm mutants that are sensitive while their wild type resist, the susceptibilities to *B. amyloliquefaciens* metabolites of *B. pseudomallei* were tested in the planktonic but not the biofilm forms. Therefore, the consequences of interrupted genes in the biofilm formation pathway (Taweechaisupapong et al. 2005) rather than the loss of biofilm itself may play a role in the susceptibility of the mutants. Further analysis of these mutants may help understand the mechanism of these metabolites.

Partial characterization of *B. amyloliquefaciens* metabolites showed the antimicrobial activity from KKU1 was abolished when treated with proteinase K and was also heat and pH stable. This was similar to the properties reported in several antimicrobial peptides and bacteriocin**-**like substances from *Bacillus* spp. (Hammami et al. 2009; Sutyak et al. 2008). The proteins in native gels from clear zone positions when extracted and separated on SDS-PAGE showed a band of small peptides that were less than 6 kDa and a thin band at the 19 kDa position. KKU1 may produce more than one active metabolites similar to *B. amyloliquefaciens* FZB42 that was reported to produce two ribosomal proteins, plantazolicin (Scholz et al. 2011) and amylocyclicin (Butcher and Helmann 2006). Different species of *Bacilli* produced a variety of antimicrobial metabolites with different modes of action (Sumi et al. 2015). The SEM observations on *B. pseudomallei* and *E. coli* membranes that were treated with the KKU1 precipitated protein showed membrane destruction. Information from bacteriocins which are cationic peptides that display hydrophobic or amphiphilic properties target the bacterial membrane and cause pore formation (Stein 2005) or complete disintegration of the cell wall (Huang et al. 2009). Translocated peptides were also reported to alter cytoplasmic membrane septum formation, inhibit cell-wall synthesis, nucleic-acid synthesis, protein synthesis or enzymatic activity (Brogden 2005). The precipitated proteins from KKU1 could alter membrane septum formation at low concentrations as seen from Gram’s stain cell morphology (data not shown) and then membrane destruction at higher concentrations as seen from the SEM images. The MIC and MBC of the precipitated proteins from KKU1 against *B. pseudomallei* (0.97 μg/ml and 3.9 μg/ml) were lower than ceftazidime (2 μg/ml and 4 μg/ml) that drawn attention for further characterization.

In conclusion, the metagenomics approach here demonstrated a higher diversity of microbes in soils that were negative for *B. pseudomallei* and for the first time described phyla that were significantly found higher in both positive and negative soils. Intensive investigation in the negative soil could lead to discovery of *B. amyloliquefaciens* isolates producing a broad spectrum of secondary metabolites that are active against *B. pseudomallei* and other Gram-negative pathogens but not the Gram-positive bacteria. The metabolites showed a highly potential opportunity to discover new broad-spectrum antibiotics for the treatment of melioidosis and also other Gram-negative infections.

## **Additional files**

**Additional file 1: Figure S1.** Operational taxonomic unit (OTU) accumulation curves of soil. The OTU of soil with *B. pseudomallei* (Black line) and without *B. pseudomallei* (Gray line). The observed numbers of OTUs were calculated at 3% dissimilarity.

**Additional file 2: Figure S2.** The distribution of 16S rDNA sequences across bacterial class levels in negative and positive soils.

**Additional file 3: Figure S3.** The frequencies of major class levels between positive and negative soil samples. Light gray bars represent negative soil and dark gray bars represent positive soil. Asterisks (*= *p*<0.05, ** = *p*<0.01) indicate the taxa that are significantly different in relative abundances of negative soil and positive soil samples.

**Additional file 4: Table S1.** Antimicrobial activity of supernatants from *B. amyloliquefaciens* against *Burkholderia* spp.

**Additional file 5: Table S2.** Antimicrobial activities of supernatants from *B. amyloliquefaciens* against *B. pseudomallei* mutants.

**Additional file 6: Table S3.** Antimicrobial activities of culture supernatants from *B. amyloliquefaciens* KKU1 and KKU14 against other pathogenic bacteria.

**Additional file 7: Table S4.** Effects of pH on the inhibitory activity of *B. amyloliquefaciens* KKU1 against *B. pseudomallei*.

## Abbreviations

LB: Luria–Bertani
MHA: Mueller-Hinton agar
MIC: minimum inhibitory concentration
MBC: minimum bactericidal concentration
OTUs: operational taxonomic units
PCR: polymerase chain reaction
