# Supplementary figures and images for: Burkholderia pseudomallei-absent soil bacterial community results in secondary metabolites that kill this pathogen
Source: AMB Express. 2018 Aug 24;8:136. doi: 10.1186/s13568-018-0663-7 (PMC6109036; doi:10.1186/s13568-018-0663-7)

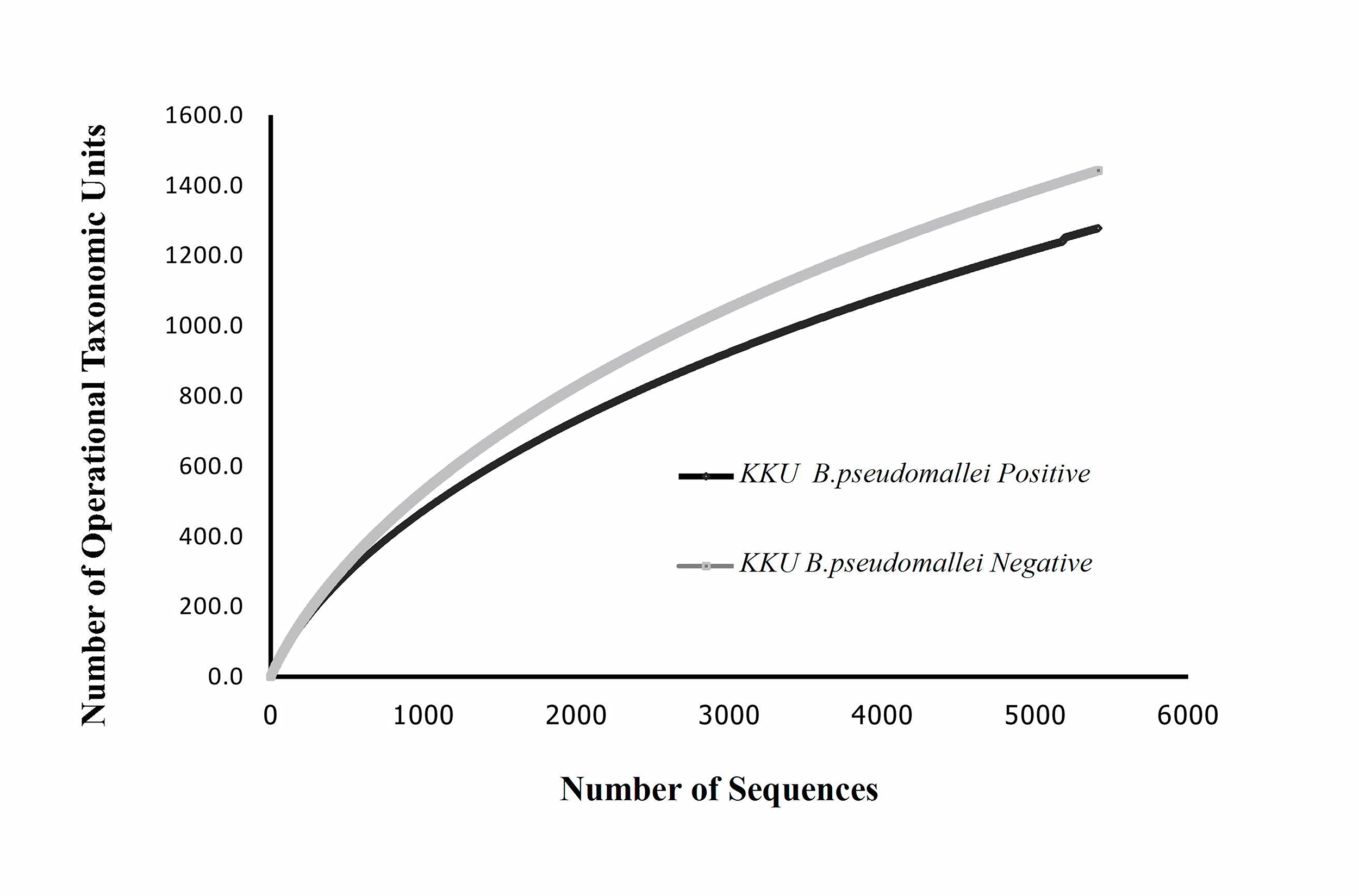

Supplement: Supplementary file 1 — Additional file 1: Figure S1. Operational taxonomic unit (OTU) accumulation curves of soil. The OTU of soil with B. pseudomallei (Black line) and without B. pseudomallei (Gray line). The observed numbers of OTUs were calculated at 3% dissimilarity. [file 13568_2018_663_MOESM1_ESM.tif]

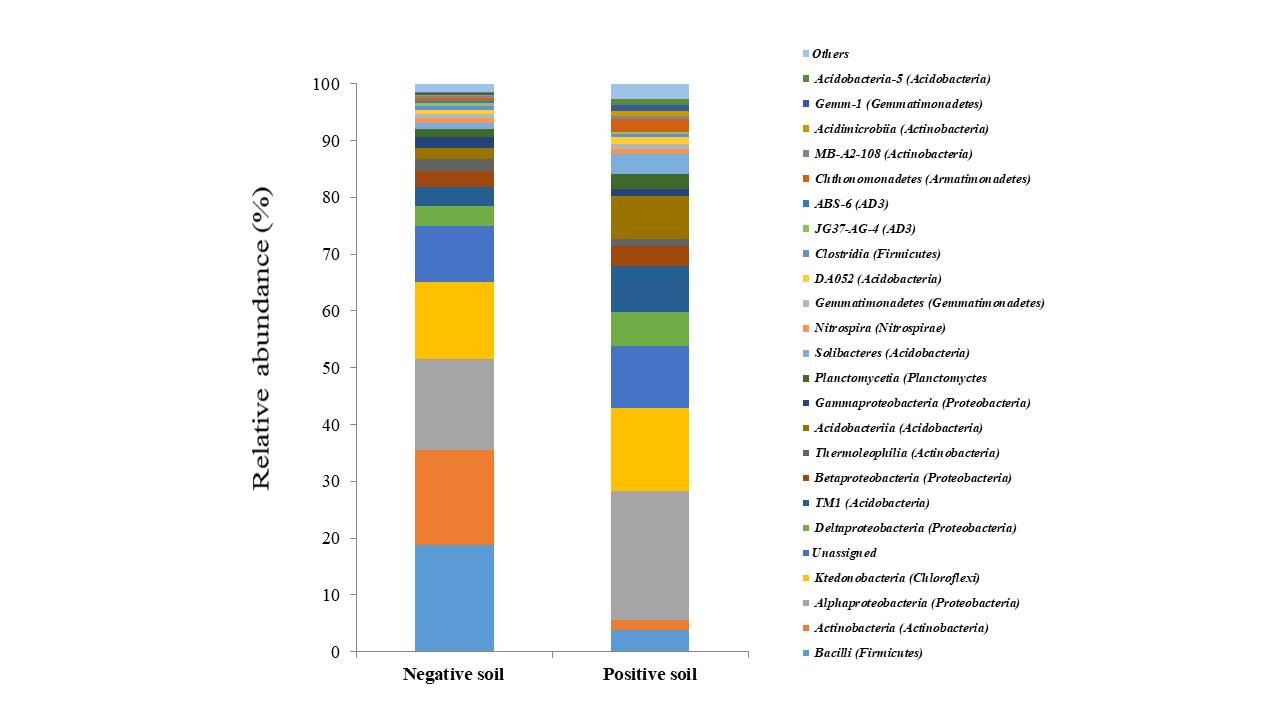

Supplement: Supplementary file 2 — Additional file 2: Figure S2. The distribution of 16S rDNA sequences across bacterial class levels in negative and positive soils. [file 13568_2018_663_MOESM2_ESM.jpg]

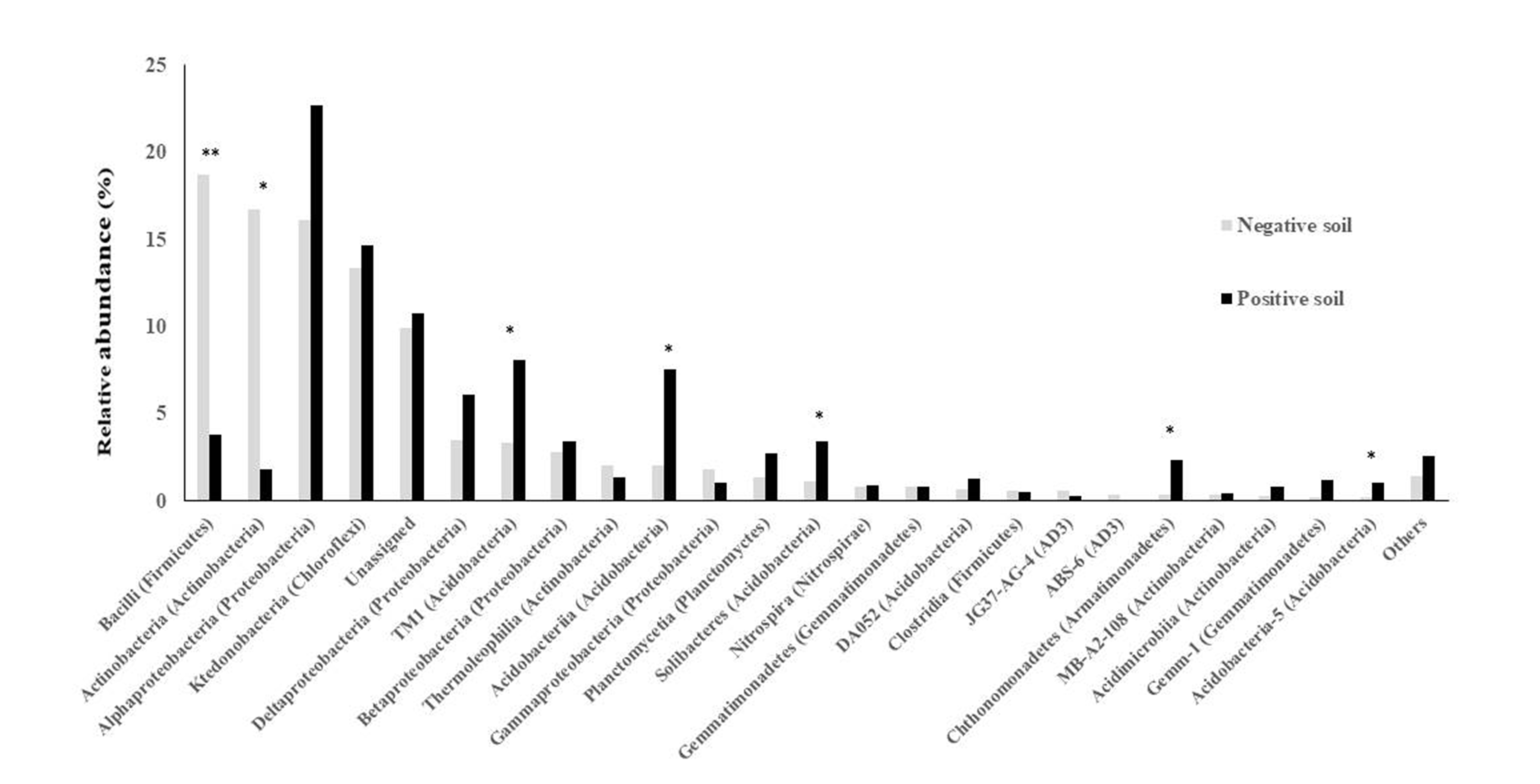

Supplement: Supplementary file 3 — Additional file 3: Figure S3. The frequencies of major class levels between positive and negative soil samples. Light gray bars represent negative soil and dark gray bars represent positive soil. Asterisks (*= p<0.05, ** = p<0.01) indicate the taxa that are significantly different in relative abundances of negative soil and positive soil samples. [file 13568_2018_663_MOESM3_ESM.tif]
