# Supplementary material for: Burkholderia pseudomallei-absent soil bacterial community results in secondary metabolites that kill this pathogen
Source: AMB Express. 2018 Aug 24;8:136. doi: 10.1186/s13568-018-0663-7 (PMC6109036; doi:10.1186/s13568-018-0663-7)
Supplement: Supplementary file 4 — Additional file 4: Table S1. Antimicrobial activity of supernatants from B. amyloliquefaciens against Burkholderia spp. [file 13568_2018_663_MOESM4_ESM.docx]

**Additional file 4: Table S1.** Antimicrobial activity of supernatant from *B. amyloliquefaciens* against *Burkholderia* spp.

| **Bacteria** | **Type** | Inhibition zone (mm) | | | |
| --- | --- | --- | --- | --- | --- |
|  |  | KKU1 | KKU3 | KKU11 | KKU14 |
| *B.pseudomallei* 316c | Drug resistant | **-** | **-** | - | 14 |
| *B.pseudomallei* 365a | Drug resistant | 18 | - | 17 | 18 |
| *B.pseudomallei* EPMN159 | Drug resistant | 13 | 15 | - | - |
| *B.pseudomallei* 979B | Drug resistant | - | - | - | - |
| *B.pseudomallei* H777 | Clinical | - | - | - | - |
| *B.pseudomallei* EPMK31 | Clinical | - | - | - | - |
| *B.pseudomallei* EPSK15 | Clinical | - | - | - | - |
| *B.pseudomallei* EPSN41 | Clinical | - | - | - | 15 |
| *B.pseudomallei* EPNL7 | Clinical | - | - | - | 13 |
| *B.pseudomallei* EPKKU27 | Clinical | 14 | - | 15 | 14 |
| *B.pseudomallei* EPUT239 | Clinical | - | - | - | 18 |
| *B.pseudomallei* EPKKU11 | Clinical | - | - | 15 | - |
| *B.pseudomallei* EPKKU15 | Clinical | - | - | - | - |
| *B.pseudomallei* EPKKU32 | Clinical | - | - | - | - |
| *B.pseudomallei* 1026b | Clinical | 13 | 13 | 15 | 17 |
| *B.pseudomallei* Zsk1 | Environmental | 14 | 13 | - | 14 |
| *B.pseudomallei* Zsk2 | Environmental | - | - | - | 13 |
| *B.pseudomallei* Zsk4 | Environmental | 14 | 17 | - | 15 |
| *B.pseudomallei* Zsk6 | Environmental | - | - | - | 16 |
| *B.pseudomallei* Zsk7 | Environmental | 18 | 15 | - | 18 |
| *B.pseudomallei* Zsk8 | Environmental | 17 | 12 | - | 18 |
| *B.pseudomallei* Zsk9 | Environmental | 19 | 18 | - | 18 |
| *B.thailandensis* MBEP159 |  | - | - | - | - |
| *B.thailandensis* MBEP164 |  | - | - | - | - |
| *B.thailandensis* MBEP167 |  | - | - | - | - |
| *B.thailandensis* MBEP169 |  | - | - | - | - |
| *B.thailandensis* MBEP170 |  | - | - | - | - |
| *B.cepacia* D1 |  | - | - | - | - |
| *B.cepacia* WW 2-1 |  | - | - | - | - |
| *B.cepacia* WW 2-2 |  | - | - | - | - |
| *B.cepacia* WW 2-4 |  | - | - | - | - |
| *B.mallei* Ey2238 |  | - | - | - | - |
| *B.mallei* Ey2236 |  | 18 | 15 | - | 17 |
| *B.mallei* Ey100 |  | 17 | 14 | - | 17 |

- indicated no inhibition zone
