# Supplementary material for: Burkholderia pseudomallei-absent soil bacterial community results in secondary metabolites that kill this pathogen
Source: AMB Express. 2018 Aug 24;8:136. doi: 10.1186/s13568-018-0663-7 (PMC6109036; doi:10.1186/s13568-018-0663-7)
Supplement: Supplementary file 5 — Additional file 5: Table S2. Antimicrobial activities of supernatants from B. amyloliquefaciens against B. pseudomallei mutants. [file 13568_2018_663_MOESM5_ESM.docx]

**Additional file 5: Table S2.** Antimicrobial activity of supernatant from *B. amyloliquefaciens* against *B. pseudomallei* mutants.

| **Strains** | **Phenotypic defects** | **Inhibition zone (mm)** | | | |
| --- | --- | --- | --- | --- | --- |
|  |  | **KKU1** | **KKU3** | **KKU11** | **KKU14** |
| 1026b | Wild type | 13 | 13 | 15 | 17 |
| SR1015 | Capsule | - | - | - | - |
| SRM117 | LPS  (o-side chain moiety) | - | - | - | - |
| MM35 | flagellin | 16 | 18 | - | 15 |
| H777 | Wild type | - | - | - | - |
| M10 | Biofilms | 19 | 20 | - | 18 |
| M6 | Biofilms | 13 | 13 | - | 14 |

- indicated no inhibition zone
