# Supplementary material for: Burkholderia pseudomallei-absent soil bacterial community results in secondary metabolites that kill this pathogen
Source: AMB Express. 2018 Aug 24;8:136. doi: 10.1186/s13568-018-0663-7 (PMC6109036; doi:10.1186/s13568-018-0663-7)
Supplement: Supplementary file 6 — Additional file 6: Table S3. Antimicrobial activities of culture supernatants from B. amyloliquefaciens KKU1 and KKU14 against other pathogenic bacteria. [file 13568_2018_663_MOESM6_ESM.docx]

**Additional file 6: Table S3.** Antimicrobial activity of culture supernatant from *B. amyloliquefaciens*  KKU1 and KKU14 against other pathogenic bacteria.

| **Bacteria** | **Gram** | **Inhibition**  **zone (mm)**  KKU1 KKU14 | |
| --- | --- | --- | --- |
| *Stenotrophomonas maltophilia* | Gram-negative | 20.5±1 | 23.7±0 |
| *Klebsiella pneumoniae* | Gram-negative | - | - |
| *Acinetobacter baumannii* | Gram-negative | - | - |
| *Salmonella* group D | Gram-negative | 14±0 | 18±0 |
| *Pseudomonas aeruginosa* | Gram-negative | 12±0 | 12±0 |
| *Shigella* group D | Gram-negative | 30±0 | 20±0.5 |
| *Escherichia coli* | Gram-negative | 12±0 | 16±0 |
| *Vibrio parahaemolyticus*  *Proteus vulgaris*  *Staphylococcus* spp*.*  *Enterococcus* spp.  *Streptococcus* spp. | Gram-negative  Gram-negative  Gram-positive  Gram-positive  Gram-positive | 17±0  15±0  **-**  **-**  **-** | 17±0  23±0  -  -  - |

* The diameters are means ±SD from duplicate experiments.
