# Supplementary material for: Burkholderia pseudomallei-absent soil bacterial community results in secondary metabolites that kill this pathogen
Source: AMB Express. 2018 Aug 24;8:136. doi: 10.1186/s13568-018-0663-7 (PMC6109036; doi:10.1186/s13568-018-0663-7)
Supplement: Supplementary file 7 — Additional file 7: Table S4. Effects of pH on the inhibitory activity of B. amyloliquefaciens KKU1 against B. pseudomallei. [file 13568_2018_663_MOESM7_ESM.docx]

**Additional file 7: Table S4.** Effects of pH on the inhibitory activity of *Bacillus amyloliquefaciens* KKU1 against *B. pseudomallei*.

| **pH** |  | **Clear Zone**  **(mm.)** |
| --- | --- | --- |
| 2 |  | 13±0.0 |
| 4 |  | 17±0.5 |
| 6 |  | 19±1.0 |
| 7 |  | 19±0.0 |
| 8 |  | 18±0.0 |
| 12 |  | 13±0.5 |
| Supernatant (8.34) |  | 20±0.0 |
| LB (6.75) |  | - |

Results shown are the average of ±SD of experiments performed in duplicate.
